# Improvement of Adhesive Wear Behavior by Variable Heat Treatment of a Tool Steel for Sheet Metal Forming

**DOI:** 10.3390/ma12172831

**Published:** 2019-09-03

**Authors:** Alejandro Gonzalez-Pociño, Florentino Alvarez-Antolin, Juan Asensio-Lozano

**Affiliations:** Materials Pro Group, Departamento de Ciencia de los Materiales e Ingeniería Metalúrgica, Universidad de Oviedo, Independencia 13, 33004 Oviedo, Spain

**Keywords:** tool steel, powder metallurgy, adhesive wear, nitriding, carbonitrides, tempering

## Abstract

Vanadis 10 steel is a powder metallurgy (PM) processed tool steel. It is a ledeburitic steel with 8% Cr and 10% V. By deliberately varying the process parameters related to the quenching, tempering, and nitriding of these steels, the aim of this study is to determine which of these parameters have a significant influence on its adhesive wear resistance. The research methodology employed was a Design of Experiments (DoE) with six factors and two levels for each factor. The tempering temperature, number of temperings, and carrying out of a thermochemical nitriding treatment were found to have a significant effect. To increase adhesive wear resistance, austenitization at 1100 °C with air cooling is recommended, followed by three temperings at 500 °C and a subsequent nitriding treatment. It should be noted that the quench cooling medium does not have a significant influence on wear resistance. Furthermore, (Fe,Cr)_7_C_3_ (M_7_C_3_ carbides) are transformed into carbonitrides during nitriding. However, (Fe,V)C (MC carbides) are not affected by this nitriding process.

## 1. Introduction

Vanadis 10 steel is a powder metallurgy (PM) processed tool steel marketed by the UDDEHOLM Company (Hagfors, Sweden). This steel is provided in the annealed condition and in order to provide the needed service properties it requires further treatments to be applied consisting of austenite destabilizing, followed by quenching and further tempering. Being a tool steel for cold working an optional surface treatment to increase the tool life is commonly applied by nitriding [1]. Table 1 gives its chemical composition. It is a ledeburitic steel alloyed mainly with Cr and V. These steels are used to manufacture tooling used to shape materials and are commonly used as dies in processes of drawing, extrusion, and forming, both in the forming of low carbon steels and in the forming of Al alloys [2]. The microstructure of this steel presents two types of carbides: MC, mainly associated with Vanadium, and M_7_C_3_, formed mainly by Chromium [3]. The usual heat treatment consists of high temperature austenitizing in which the M_7_C_3_ carbides and a small part of the MC carbides are redissolved, the latter being more stable and more averse to dissolving in the austenite. Dissolution of the Cr and part of the V leads to an increase in the hardness of the material after quenching in oil [4,5]. The undissolved carbides hinder austenite grain growth and afford hardness and a high wear resistance to the steel [6]. By destabilizing the austenite via holding times of more than four hours at the austenitizing temperature [7], two effects are achieved: (1) The stabilization of supersaturated austenite, which accomplishes the precipitation of secondary carbides M_7_C_3_, and (2) the decrease in the percentage of retained austenite after quenching. The latter would be caused by an increase in the temperature of the onset of martensitic transformation (Ms) because the austenite would be less alloyed by the precipitation of said carbides [8]. After quenching, Vanadis 10 steel is mainly composed of martensite, retained austenite, and MC and M_7_C_3_ carbides [3]. The austenite is difficult to transform into martensite, although it is possible to promote this transformation on tempering at temperatures above 470 °C [9,10]. The retained austenite practically disappears in its entirety after a double tempering at 550 °C [11]. During tempering, a fine dispersion of nanometric carbides occurs in the martensitic structure leading to secondary hardening. This effect is strongly related to the amount of carbide forming elements (V, Mo or Cr) remaining in solid solution, which is connected to the destabilization treatment of austenite. At higher ageing temperatures, the carbides coarsen, increasing in size, which leads to a reduction in the hardness of the alloy [11]. In cold forming processes, which constitute the main use of the material under study, the principal source of heat is produced by friction [12]. These temperatures affect the surface layer of the material [13,14], allowing rapid oxidation of the material and leading to the formation of a compacted oxide film that may affect the wear rate [15]. The adhesive wear resistance of tool steels manufactured by powder metallurgy depends not only on their hardness but also on the composition of the nitrided layer and its thickness [16]. The steels to be nitrided should always be quenched and tempered. The tempered martensite favors the diffusion of N and contributes toughness to the central nucleus, which means it is able to resist the major stresses that the nitrided outer layer will transmit to it [17]. Thermo-chemical nitriding treatments produce surface hardening via the formation of subnitrides in the tempered martensite matrix, thereby promoting an increase in the wear resistance of this type of steel [16,18,19,20,21].

This paper aims to study the effect of the different process variables related to heat treatments that may condition the adhesive wear resistance of Vanadis 10 steel. Specifically, different parameters related to the destabilization of austenite (to promote precipitation of secondary carbides), different quench cooling media, different parameters related to martensite tempering (to promote secondary hardening), and the effect of nitriding treatment are analyzed. The results thus obtained will enable tooling and die manufacturers that use this steel in the annealed state as a raw material to define the most suitable heat treatment to optimize the service behavior of the steel.

## 2. Materials and Methods

The purpose of applying a Design of Experiments (DoE) was to modify certain working conditions related to heat treatments thus to produce changes in the adhesive wear resistance of the steel under study. The analysis of these changes will enable us to determine which of the working parameters have a significant effect on this wear resistance. Table 2 shows the analyzed working parameters and the values they were assigned thus to modify the working conditions in an orderly manner. In this case, 6 factors were analyzed, with 2 levels for each factor. The effect of a factor was the variation in the response function as a consequence of the variation of said factor. These effects were defined as the main effects. If the DoE were factorial, it would be necessary to execute a total of 2^6^ (64) experiments, in which case the experiment would be carried out with all possible combinations of factors and levels. In most cases, however, the effect of one factor depends on the value that another takes. When this occurred, these factors were said to interact. The influence of the main effects tended to be greater than that of the interactions of 2 factors, while the influence of the latter was in turn greater than that of the interactions of 3 factors, and so on. Interactions of 3 or more factors with a significant effect on a given response did not appear in industrial practice [22]. That was, sufficiently approximate models were obtained considering only the main effects and the 2-factor interactions, thus allowing the number of experiments to be reduced. Fractional designs allowed studying a larger number of factors with fewer experiments compared to the corresponding saturated DoE, and were of frequent use under any of the following scenarios:

- When the number of experiments exceeds the available resources,
- When it is only required the information of the principal effects or the information provided by low order interactions of the factors,
- In exploratory studies where there are many factors,
- When the assumption is made that only a few effects are important.

Yates’ algorithm allows the calculation of the effects in a simple and systematic way and can be implemented on an electronic spreadsheet [22].

In the case in hand, 8 experiments were carried out, thus we only estimated 8 effects (2^6−3^), which supposed a 1/8 (64/8 = 8) fractional factorial design. Table 3 shows the resulting array of experiments. The set of generators associated with this array of experiments was D = AB, E = AC, and F = BC [21]. That is, columns D, E, and F have been, respectively, constructed from the product of columns A × B, A × C and B × C. The “Restricted Confounding Pattern” column indicates only the main effects and those 2-factor interactions whose effects were confounded with the main effects. The most practical method to determine if a factor (temperature, holding time, etc.) had a significant effect on the response of interest (for example: Hardness, wear resistance, etc.) was to represent the effects for a given factor in normal probability paper (y-axis: Probability percentage scaled from 0% to 100%; and x-axis: The effects of the factors that can be either negative or positive). The effects were linear combinations of the analyzed responses. Hence, applying the central limit theorem (CLT), they followed a normal law. If all the effects were non-significant, they would follow an N(0,σ) law and would thus appear aligned in a representation of the effects on a normal probability plot. Thus, factors that do not have a significant influence on the analyzed response will lie on the straight line passing through the point of coordinates (0, 50). If any effect was significant, however, it will follow an N(μ,σ) law, not appearing aligned with the non-significant effects. Those effects that deviated from the straight line towards the ends on the normal probability plot were considered significant [22]. For instance, when the effect of a factor appeared misaligned with respect to the aforementioned straight line but located at the lower end and to the left side of such line, would indicate that the factor (temperature, etc.) would increase the value of the response (hardness), if it takes a value corresponding to its −1 level. Reciprocally, if the effect deviated at the upper end and to the right side of the line, will mean that the factor would increase the value of the response when it takes a value corresponding to its +1 level.

The material was supplied in the form of 46 mm diameter bars. The state of supply was the annealed condition. The bar was cut to give billets of 25 mm length to serve as samples for the experimental work. Different heat treatments were carried out on these samples. For metallographic inspection, they were further cut and bakelite mounted, followed by mechanical grinding in SiC sandpaper of 240, 320, 400, and 600 grit. Textile cloths with 6 and 1 micron diamond paste were used during the subsequent mechanical polishing. For final observation, the samples were further etched with nital 5 (5 mL nitric acid and 95 mL ethanol). The microstructures of the samples were analyzed under a NIKON Epiphot 200 inverted optical microscope (Nikon, Tokyo, Japan). The scanning electron microscope employed (SEM) was a JEOL JSM-5600 (JEOL, Nieuw-Vennep, Netherlands), equipped with the characteristic energy dispersive X-ray (EDX) microanalysis system. The adhesive wear tests were performed on a Pin on Disc tribometer, according to the ASTM G99 standard, using a Micro-Test MT/30/SCM/T device (MicroTest, Madrid, Spain) for this purpose. The nitriding process was carried out with dissociated NH_3_ in two stages: In a first stage, employing a dissociation degree of 25% at 520 °C for 8 h; and in a second stage, employing a dissociation degree of 60% at 540 °C for 14 h.

**Figure 1:**
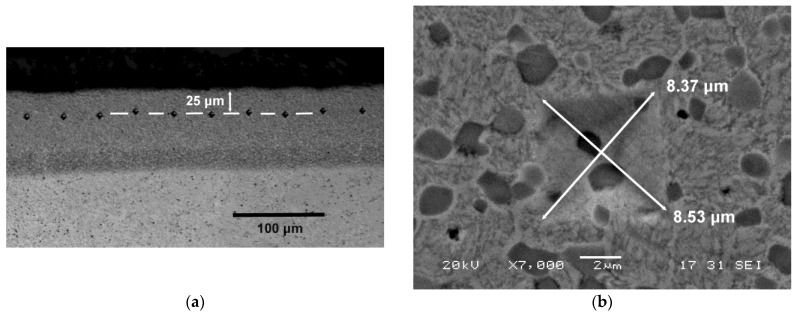
A total of 20 indentations were made for each experiment. (**a**) All the indentations were placed at 25 μm from the outer edge of the nitrided layer; (**b**) The measurements were determined at 7000× on a scanning electron microscope (SEM) images.

The analyzed responses were:

- The Vickers hardness before nitriding. The applied load was of 294.2 N, while the value considered in each experiment was the average value obtained from 10 hardness indentations.
- The Vickers hardness of the nitrided layer in Experiments 5 to 8. The applied load was 0.5 N. The estimated hardness value was made to coincide with the average value obtained from the 20 indentations made at 25 μm from the outer face of the nitrided layer (see depiction in Figure 1).
- The adhesive wear resistance by means of the pin on disc test with a linear speed of 0.38 ms^−1^ and a load of 30 N. The pin corresponded with each of the 8 experiments. It had a circular cross-section of 3 mm in diameter. The disk was manufactured in steel manufactured in accordance with the DIN 42CrMo4 (AISI 4140) standard in the oil-quenched state. Its hardness at the time of the trials was 650 HV.

## 3. Results and Discussion

**Figure 2:**
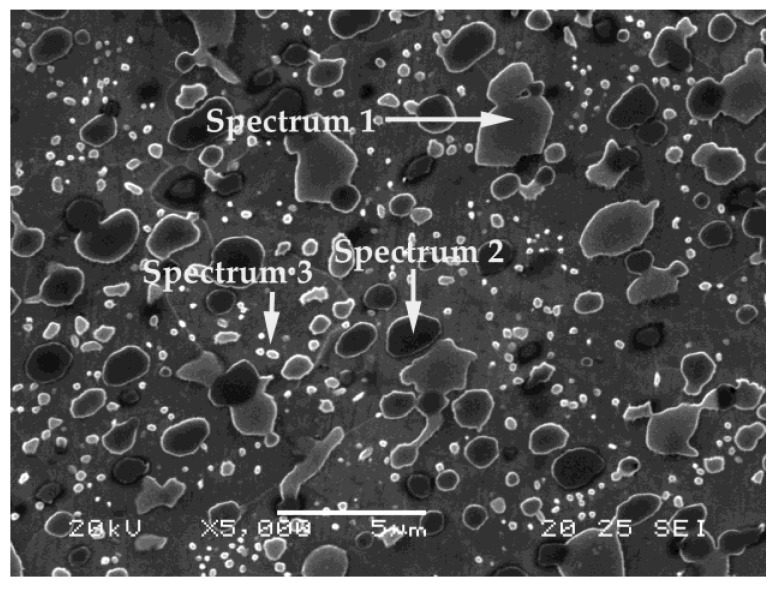
Microstructure of Vanadis 10 steel in the annealed state.

Figure 2 shows the representative structure of the Vanadis 10 steel in the annealed state. A high density of carbides can be seen in a ferritic matrix. Arrows are used to indicate the carbides that were analyzed semi-quantitatively by energy-dispersive X-ray (EDX) microanalysis. Table 4 shows the results thus obtained.

The carbide corresponding to Spectrum 1 is associated with M_7_C_3_, while the carbide corresponding to Spectrum 2 is associated with MC. The shiny carbides, with a globular appearance and a size of below 1 μm, corresponding to Spectrum 3, could be associated with M_3_C-type cementite carbides. These would correspond to the eutectoid constituent. Eutectic carbides of the type M_7_C_3_ and MC are the only ones that include Mo atoms.

The phases present after the quenching and tempering treatments were tempered martensite, retained austenite and M_7_C_3_ and MC carbides [3]. Some M_2_C carbides associated with Mo may also be identified [3].

**Figure 3:**
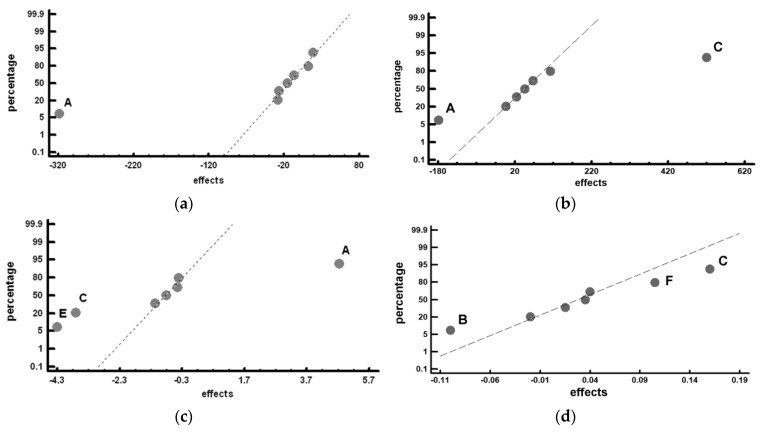
Representation of the effects on a normal probability plot. (**a**) measured hardness before the nitriding treatment; (**b**) measured hardness including the nitriding treatment in Experiments 5 to 8; (**c**) weight loss after the wear tests; (**d**) coefficient of friction in the wear test.

Table 5 shows the mean values obtained in each experiment, together with the effects corresponding to the restricted confounding pattern. Figure 3 shows the representation of these effects on a normal probability plot, highlighting those that have a significant effect on these responses.

Figure 3a analyses the effect of the factors on hardness but does not include factor C (nitriding treatment). That is, it analyses the effect of the factors on the hardness of the material, though before carrying out the nitriding treatment. It can be seen that the only factor with a significant effect was the tempering temperature (Factor A). Thus, if the aim was to increase the hardness of the steel, this factor should be placed at its −1 level (tempering at 500 °C). The technical literature confirms that this strengthening mechanism corresponds to secondary hardening, a process that occurred by the precipitation of nanometric Cr_7_C_3_ carbides at a temperature of 500 °C [23,24]. Cr remains in solid solution at the first stages of martensite ageing. It is only when the temperature reaches 500 °C approximately, that Cr reaches its solubility product for precipitation to form stable carbides on ageing. At this stage, strengthening by secondary hardening occurs increasing the steel stress needed to yielding. Technical literature [24] upholds that gradually increasing the Cr content, in the presence of V and Mo, induces higher peak strengths that remain constant at a relatively short range of temperatures. In addition, these observations corroborate that the higher the Cr content the further the tendency to carbide overaging, which imposes an upper limit to the ageing temperature in order to gain the optimum strength. The former supports that temperatures in excess of 600 °C will lead to a severe drop in hardness from peak value.

Figure 3b shows the factors with a significant effect on hardness following the nitriding treatment. As indicated in Table 3, this treatment was only carried out in Experiments 5 to 8. It can be seen that Factors A (tempering temperature) and C (nitriding treatment) have a significant effect. Thus, in order to increase the hardness of the steel, these factors should be placed at their −1 (tempering at 500 °C) and +1 (carrying out of the nitriding treatment) levels, respectively.

Figure 3c shows the factors with a significant effect on adhesive wear resistance. These factors are: A (tempering temperature), C (nitriding treatment), and E (number of temperings). Similar to the previous case, in order to increase the resistance, the adhesive wear, these factors should be placed at the same levels as those specified to increase the material’s hardness. That is, at levels −1 (tempering at 500 °C), +1 (carrying out of the nitriding treatment) and +1 (3 tempers), respectively. Performing three tempers could promote a reduction in retained austenite to practically zero levels [3].

Figure 3d shows the factors with a significant effect on the coefficient of friction. The coefficient increases when placing Factor B at its −1 level (4 h at 1100 °C) and Factors C and F at their +1 level (nitriding treatment and tempering times of 4 h). Factor B at its +1 level (8 h holding time at 1100 °C) leads to an increase in the volume fraction of MC carbides [3]. It may thus be concluded that MC carbides decrease the coefficient of friction, without thereby affecting wear resistance. The nitriding treatment is also seen to increase the coefficient of friction. In this case, however, this factor increases the adhesive wear resistance of the material, as seen above.

**Figure 4:**
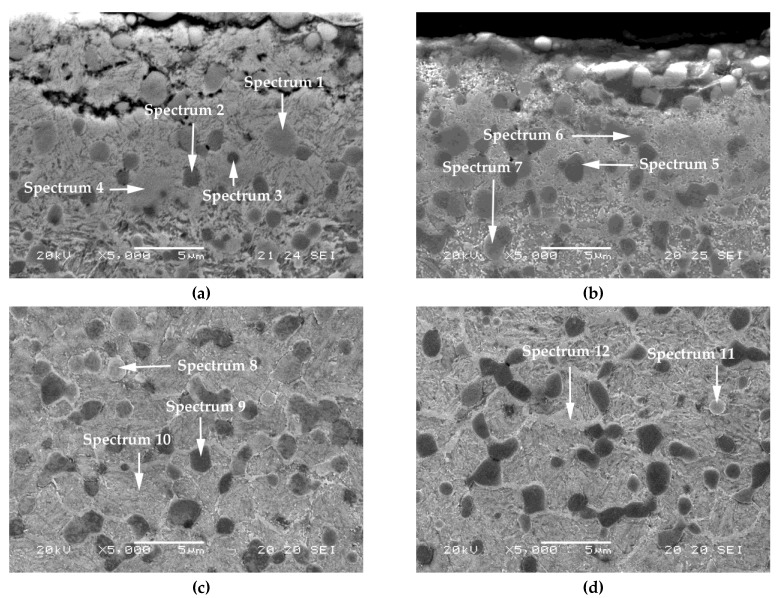
Phases of the nitrided layer, analyzed by energy dispersive X-ray (EDX) microanalysis; (**a**) Experiment 5; (**b**) Experiment 6; (**c**) Experiment 7; (**d**) Experiment 8.

Figure 4 provides a representative image of the nitrided layer in Experiments 5 to 8. These micrographs show the phases of the nitrided layers analyzed semi-quantitatively by characteristic X-ray scattering (EDX) microanalysis, the results of which are given in Table 6. Figure 4a shows the presence of cracks in the outer edge of the nitrided layer, which could be formed in the cutting process during the preparation of the metallographic specimens. It should be noted that, following the nitriding treatment, the M_7_C_3_ carbides were transformed into carbonitrides (Spectra 1, 6, 7, 8, and 11). In all cases, the percentages in weight of nitrogen exceed 15%. However, the MC carbides were not affected by this nitriding process (Spectra 2, 3, 5, and 9). Furthermore, the matrix constituent of this nitrided layer reached values of between 5 and 8 wt.% N, denoting a lower enrichment in N (Spectra 4, 10, and 12). Due to the low solubility of N in Fe and its high affinity for Cr, it is reasonable to expect the presence of Cr nitrides in the tempered martensite in addition to the nitrides associated with the Fe-N system.

## 4. Conclusions

From the analysis of the process parameters related to the heat treatments of the Vanadis 10 steel, it is concluded that in order to increase the material’s adhesive wear resistance, the following heat treatment can be recommended:

1. The results show that the quench cooling medium after destabilization of austenite at 1100 °C seems not to have a significant influence on wear resistance. This allows manufacturers to quench tools in the air with the corresponding savings as well as to reduce the risk of quench cracking.
2. In regards to the holding time at the destabilizing temperature of 1100 °C, it can be concluded that long holding times will not yield a significant effect on adhesive wear resistance.
3. Our findings show that the most suitable tempering temperature should be 500 °C for optimum performance against adhesive wear.
4. The analysis also shows that the best results are obtained when three tempering treatments of two h/each are conducted at 500 °C.
5. The nitriding treatment produces an increase in the resulting hardness values comprised between 350 and 700 HV with regard to the samples without thermo chemical treatment.
6. Our observations show that the M_7_C_3_ carbides are transformed into carbonitrides during nitriding, with variable weight percentages of above 15 wt.%. However, MC carbides seemed not to be affected by this nitriding process. The weight percentage of N in the matrix constituent in the nitrided layer lies between five and eight wt.%.

## Figures and Tables

**Table 1:** Composition (wt.%).

C	Si	Mn	Cr	Mo	V
2.9	0.5	0.5	8	1.5	9.8

**Table 2:** Factors and levels. Heat-up rates of about 15 °C per minute and cool down rates on quenching in the range of 70–80 °C per second were used.

Factors			Levels	
Code	Description of The Factors	Units	−1 Level	+1 Level
A	Tempering temperature	°C	500	600
B	Holding time at 1100 °C	h	4	8
C	Nitriding	-	No	Yes
D	Quench cooling medium	-	air	oil
E	Number of temperings	-	2	3
F	Tempering time	h	2	4

**Table 3:** Array of experiments.

No.	A	B	C	D	E	F	Restricted Confounding Pattern
1	−1	−1	−1	+1	+1	+1	A + BD + CE B + AD + CF C + AE + BF D + AB + EF E + AC + DF F + BC + DE AF + BE + CD
2	+1	−1	−1	−1	−1	+1	
3	–1	+1	−1	−1	+1	−1	
4	+1	+1	−1	+1	−1	−1	
5	−1	−1	+1	+1	−1	−1	
6	+1	−1	+1	−1	+1	−1	
7	−1	+1	+1	−1	−1	+1	
8	+1	+1	+1	+1	+1	+1	

**Table 4:** Analysis of the phases listed in Figure 2, determined by the characteristic energy dispersive X-ray (EDX) microanalysis. Results are presented in atomic %.

Spectrum	%C	%V	%Cr	%Fe	%Mo	Most Likely Carbide Type
1	67.1	4.38	15.01	13.11	0.40	M_7_C_3_
2	62.29	20.19	4.60	11.93	0.98	MC
3	38.75	2.96	5.16	53.13	-	M_3_C

**Table 5:** Values and effects obtained for the analyzed responses.

(**a**) Hardness before nitriding.						
**Experiment**	**HV**	**Auxiliary Columns Corresponding to Yates’ Algorithm**			**Effects**	
		**I**	**II**	**III**		
1	824 ^1^	1328	2614	5303	662.8	Average
2	504 ^1^	1286	2689	−1275	−318.7	A + BC + CE
3	775 ^1^	1379	−584	−111	−27.7	B + AD + CF
4	511 ^1^	1310	−691	51	18.7	C + AE + BF
5	861 ^1^	−320	−42	75	12.7	D + AB + EF
6	518 ^1^	−264	−69	−107	−26.7	E + AC + DF
7	829 ^1^	−343	56	−27	−6.7	F + BC + DE
8	481 ^1^	−348	−5	−61	−15.2	AF + BE + CD
(**b**) Hardness after nitriding.						
**Experiment**	**HV**	**Auxiliary Columns Corresponding to Yates’ Algorithm**			**Effects**	
		**I**	**II**	**III**		
1	824 ^1^	1328	2614	7309	913.5	Average
2	504 ^1^	1286	4695	−717	−179.2	A + BC + CE
3	775 ^1^	2234	−584	185	46.4	B + AD + CF
4	511 ^1^	2461	−133	99	520	C + AE + BF
5	1161 ^2^	−320	−42	2081	24.7	D + AB + EF
6	1073 ^2^	−264	227	451	112.7	E + AC + DF
7	1253 ^2^	−88	56	269	67.4	F + BC + DE
8	1208 ^2^	−45	43	−13	−3.3	AF + BE + CD
(**c**) Pin-on-disc wear.						
**Experiment**	**Δ** **m (mg)**	**Auxiliary Columns Corresponding to Yates’ Algorithm**			**Effects**	
		**I**	**II**	**III**		
1	2.4	13.5	25.5	36.2	4.52	Average
2	11.1	12	10.7	19	4.75	A + BC + CE
3	1.3	6.9	18.1	−4.6	1.15	B + AD + CF
4	10.7	3.8	0.9	−1.8	−3.7	C + AE + BF
5	2.6	8.7	−1.5	−14.8	−0.45	D + AB + EF
6	4.3	9.4	−3.1	−17.2	−4.3	E + AC + DF
7	2.3	1.7	0.7	−1.6	−0.4	F + BC + DE
8	1.5	−0.8	−2.5	−3.2	−0.8	AF + BE + CD
(**d**) Pin-on-disc wear.						
**Experiment**	**μ**	**Auxiliary Columns Corresponding to Yates’ Algorithm**			**Effects**	
		**I**	**II**	**III**		
1	0.77	1.51	2.61	5.86	0.732	Average
2	0.74	1.1	3.25	0.16	0.04	A + BC + CE
3	0.51	1.62	0.05	−0.4	−0.1	B + AD + CF
4	0.59	1.63	0.11	0.14	0.16	C + AE + BF
5	0.79	−0.03	−0.41	0.64	0.03	D + AB + EF
6	0.83	0.08	0.01	0.06	0.01	E + AC + DF
7	0.78	0.04	0.11	0.42	0.10	F + BC + DE
8	0.85	0.07	0.03	−0.08	−0.02	AF + BE + CD

^1^ The applied load was 294.2 N. ^2^ The load applied in the nitrided layer was 0.5 N. Δm: Weight loss on the pin. µ: Coefficient of friction.

**Table 6:** Semi-quantitative analysis of the phases listed in Figure 4 by means of energy dispersive X-ray (EDX) microanalysis. (% in weight).

Spectrum	%C	%V	%Cr	%Fe	%Mo	%N
1	10.76	9.81	27.88	29.76	2.21	19.59
2	18.24	39.76	7.48	30.56	3.97	-
3	14.28	38.52	6.93	35.04	5.23	-
4	11.98	1.80	5.88	71.59	-	8.75
5	24.50	46.05	6.37	18.55	4.53	-
6	13.41	9.93	23.51	30.94	1.35	20.87
7	16.18	7.39	17.17	40.67	1.30	17.30
8	9.22	9.03	32.97	30.55	2.40	15.84
9	32.40	29.56	6.25	28.60	3.19	-
10	6.63	2.86	6.96	77.71	-	5.84
11	11.41	9.4	30.56	29.66	2.34	14.63
12	24.42	2.27	6.42	60.77	-	6.12
